# Unveiling the Impact of Organic Fertilizer on Rice (*Oryza sativa* L.) Salinity Tolerance: Insights from the Integration of NDVI and Metabolomics

**DOI:** 10.3390/plants14060902

**Published:** 2025-03-13

**Authors:** Jiaolong Li, Yunluo Li, Qiyun Xu, Xiaolei Niu, Guangping Cao, Hongyan Liu

**Affiliations:** 1 College of Tropical Agriculture and Forestry, Hainan University, Haikou 570228, China; lijiaolong@yzwlab.cn (J.L.); leexh309@gmail.com (Y.L.); xuqiyun83@gmail.com (Q.X.); 2 School of Breeding and Multiplication, Hainan University, Haikou 570228, China; 3 Institute of Food Crops, Hainan Academy of Agriculture Sciences, Haikou 571100, China; 4 Hainan Key Laboratory of Crop Genetics and Breeding, Hainan Academy of Agriculture Sciences, Haikou 571100, China

**Keywords:** salinity stress, organic fertilizer, NDVI, metabolomics, rice stress tolerance

## Abstract

Soil salinization threatens global agriculture, reducing crop productivity and food security. Developing strategies to improve salt tolerance is crucial for sustainable agriculture. This study examines the role of organic fertilizer in mitigating salt stress in rice (*Oryza sativa* L.) by integrating NDVI and metabolomics. Using salt-sensitive (19X) and salt-tolerant (HHZ) cultivars, we aimed to (1) evaluate changes in NDVI and metabolite content under salt stress, (2) assess the regulatory effects of organic fertilizer, and (3) identify key metabolites involved in stress response and fertilizer-induced regulation. Under salt stress, survival rate of the 19X plants dropped to 6%, while HHZ maintained 38%, with organic fertilizer increasing survival rate to 25% in 19X and 66% in HHZ. NDVI values declined sharply in 19X (from 0.56 to <0.25) but remained stable in HHZ (~0.56), showing a strong correlation with survival rate (R^2^ = 0.87, *p* < 0.01). NDVI provided a dynamic, non-destructive assessment of rice health, offering a faster and more precise evaluation of salt tolerance than survival rate analysis. Metabolomic analysis identified 12 key salt-tolerant metabolites, including citric acid, which is well recognized for regulating salt tolerance. HTPA, pipecolic acid, maleamic acid, and myristoleic acid have previously been reported but require further study. Additionally, seven novel salt-tolerant metabolites—tridecylic acid, propentofylline, octadeca penten-3-one, 14,16-dihydroxy-benzoxacyclotetradecine-dione, cyclopentadecanolide, HpODE, and (±)8,9-DiHETE—were discovered, warranting further investigation. Organic fertilizer alleviated salt stress through distinct metabolic mechanisms in each cultivar. In 19X, it enhanced antioxidant defenses and energy metabolism, mitigating oxidative damage and improving fatty acid metabolism. In contrast, HHZ primarily benefitted from improved membrane stability and ion homeostasis, reducing lipid peroxidation and oxidative stress. These findings primarily support the identification and screening of salt-tolerant rice cultivars while also highlighting the need for cultivar-specific fertilization strategies to optimize stress resilience and crop performance. Based on the correlation analysis, 26 out of 53 differential metabolites were significantly correlated with NDVI, confirming a strong association between NDVI shifts and key metabolic changes in response to salt stress and organic fertilizer application. By integrating NDVI and metabolomics, this study provides a refined method for evaluating salt stress responses, capturing early NDVI changes and key salinity stress biomarkers. This approach may prove valuable for application in salt-tolerant variety screening, precision agriculture, and sustainable farming, contributing to scientific strategies for future crop improvement and agricultural resilience.

## 1. Introduction

Soil salinization is one of the most pressing global challenges in agriculture. It affects approximately 20% of irrigated lands, threatening the productivity of major crops and the livelihood of millions of farmers [1]. Salinity impairs plant growth by disrupting ion homeostasis, inducing osmotic stress, and inhibiting photosynthetic processes [2]. Among staple crops, rice (*Oryza sativa* L.) is particularly sensitive to salinity stress, which can cause significant yield losses. Salinity hampers water uptake and increases ionic toxicity, leading to chlorosis, stunted growth, and ultimately, reduced grain production [3,4]. The intensification of soil salinization due to climate change and unsustainable agricultural practices has heightened the urgency of addressing this issue. Developing strategies to improve salinity tolerance in rice is therefore essential for ensuring food security in salt-affected regions [5]. Current research highlights the need for integrated approaches that combine genetic improvement with effective agronomic practices to mitigate salinity’s impacts on rice productivity.

Salinized soils often exhibit poor structure, reduced porosity, and impaired aeration and permeability, making it easier for salts to accumulate around the root zone, which severely affects crop growth [6]. Organic fertilizers, rich in organic matter, humic acids, and polysaccharides, are able to rapidly combine with soil particles after being applied, forming stable soil aggregates [7]. These aggregates not only increase porosity and permeability but also enhance the soil’s ability to buffer moisture and nutrients, thereby reducing the soil’s electrical conductivity (EC) and sodium adsorption ratio (SAR) to a certain extent [8]. Studies have shown that organic acids released during the decomposition of organic fertilizers rich in humus can exchange with Na^+^ on the surface of soil colloids, making Na^+^ more likely to be leached deep into the soil by irrigation or rainfall, thus maintaining a more favorable ion balance [9]. Moreover, many studies have emphasized the synergistic effects of organic fertilizers in improving salinized soils. When applied in combination with gypsum, lime, sulfur-containing compounds, or other soil amendments, Na^+^ in the soil colloids can be further displaced. When used in conjunction with straw return or biochar, more durable and stable aggregate structures can be formed [10]. Therefore, it is evident that organic fertilizers can not only independently improve salinized soils but can also work in combination with other techniques, maximizing the reduction of salt accumulation in the plow layer and providing a more ideal environment for rice root growth.

Salinized soils typically suffer from insufficient nutrient supply and nutrient imbalances. Meanwhile, excessive sodium ions (Na^+^) in the soil not only directly cause ion toxicity but also affect the absorption of other beneficial ions such as potassium (K^+^), calcium (Ca^2+^), and magnesium (Mg^2+^) [3]. Organic fertilizers characterized by a wide variety of nutrients and slow, sustained nutrient release are able to effectively alleviate nutrient deficiencies under salt stress conditions [11]. During the decomposition of organic fertilizers, microbial activity releases organic acids, amino acids, and other substances, which can form complexes or chelates with soil cations, making K^+^, Ca^2+^, and other nutrients more readily absorbed by rice [12]. At the same time, by promoting the displacement and leaching of Na^+^, they can create the necessary conditions for maintaining a high K^+^/Na^+^ ratio in rice[9]. Furthermore, some organic fertilizer products intentionally include calcium, magnesium, trace elements, or natural minerals, which can enhance the improvement effect by competing for binding or exchange with salt ions [13]. Therefore, from the perspective of improving salinized soils, organic fertilizers are capable of multiple effects, reducing salts while supplementing necessary macronutrients and micronutrients, thereby providing a solid nutritional foundation for rice growth.

Organic fertilizers have long been recognized for their benefits in improving soil fertility and plant health. They enhance soil structure, increase water retention, and provide essential nutrients that support plant growth [14]. In the context of salinity stress, organic fertilizers play a critical role in mitigating its adverse effects by improving soil physical properties and fostering a conducive environment for root growth [15]. Moreover, organic fertilizers are able to modulate plant metabolism, enhancing antioxidant defense systems and promoting the accumulation of osmoprotectants [16]. However, the efficacy of organic fertilizers varies across rice cultivars with different salt tolerance levels. A detailed investigation of their interactions with specific physiological and molecular processes is essential to optimize their use in salt-affected regions.

Metabolomics is a powerful tool for unraveling the molecular mechanisms underlying plant responses to environmental stress. By identifying and quantifying stress-induced metabolites, metabolomics provides insights into the biochemical pathways involved in stress adaptation. In crops, salinity stress triggers changes in primary and secondary metabolites, including osmolytes, antioxidants, and hormones [17]. These metabolites play crucial roles in maintaining osmotic balance, scavenging reactive oxygen species (ROS), and regulating energy metabolism [18]. Combining metabolomic data with physiological indicators enables a holistic assessment of plant stress responses. Recent studies have highlighted the potential of metabolomics to identify biomarkers for salinity tolerance, providing a molecular basis for developing stress-resilient crops. Furthermore, integrating metabolomics with advanced computational tools facilitates the mapping of complex metabolic networks, revealing key nodes and pathways that could be targeted for genetic or agronomic interventions.

The Normalized Difference Vegetation Index (NDVI) has become a widely adopted non-destructive tool for monitoring plant responses to environmental stress. NDVI reflects the photosynthetic efficiency and chlorophyll content of plants, making it an effective indicator of plant health. This index is particularly valuable for tracking temporal changes in plant stress responses and assessing the effectiveness of mitigation strategies [19,20]. Advances in remote sensing and imaging technologies have further enhanced the utility of NDVI, enabling precise and dynamic monitoring of crop performance under stress conditions. Its application in studies of salinity stress has revealed critical differences between tolerant and sensitive rice cultivars [21]. Integrating NDVI with other physiological and molecular analyses provides a comprehensive understanding of plant responses and offers a practical approach for evaluating agronomic interventions.

Current studies on the role of organic fertilizers in alleviating salinity stress are largely descriptive and speculative. The detailed regulatory mechanisms remain poorly understood. We hypothesize that organic fertilizer application mitigates salt stress in rice by modulating key metabolic pathways and improving physiological traits, with distinct regulatory mechanisms in salt-sensitive and salt-tolerant cultivars. This study aims to explore the effects of organic fertilizers on salinity stress in rice and uncover the underlying mechanisms. The objectives are as follows: 1. to identify differences in NDVI and changes in metabolites in the responses of salt-tolerant and salt-sensitive rice cultivars; 2. to evaluate the effectiveness of organic fertilizers in mitigating salinity stress and propose their potential mechanisms; 3. to determine the key components in organic fertilizers that contribute to alleviating salinity stress, if significant effects are observed. By linking NDVI dynamics with metabolomic changes, it offers a novel framework for assessing the effectiveness of agronomic interventions. This study integrates spectral phenotyping and metabolomics to provide insights into the regulatory roles of organic fertilizers.

## 2. Results

### 2.1. Survival Rates and NDVI Dynamics of Rice (Oryza sativa L.) Cultivars Under Different Treatments

**Figure 1:**
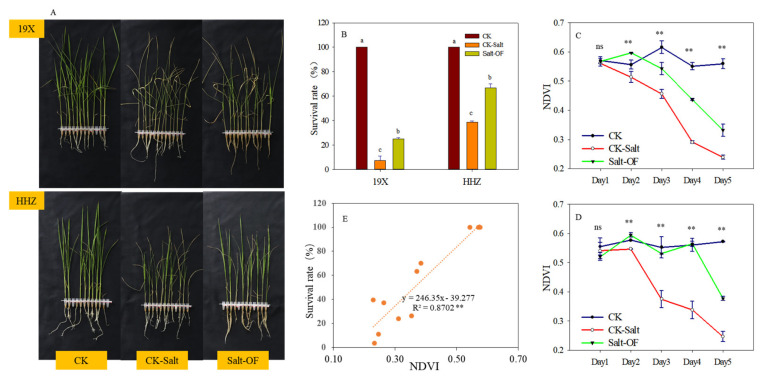
Effects of salt stress and organic fertilizer on survival rate and NDVI dynamics of two rice (*Oryza sativa* L.) cultivars. (**A**): Pictures of 19X and HHZ seedlings on Day 5 under three treatments; (**B**): Survival rate of rice seedlings of 19X and HHZ under different conditions. Error bars are S.E. of the mean of 2 independent samples from two replications. Different letters indicated significant difference at 0.05 level. (**B**,**C**): NDVI dynamics of rice seedlings of 19X (**C**) and HHZ (**D**) after treatment under different conditions. Error bars are S.E. of the mean of 2 independent samples from two replications. ** indicates significant differences (*p* < 0.01), ns indicates no significant differences at 0.05 level. (**E**): Correlation between NDVI (Day 5) and survival rate of rice seedlings of two cultivars under three treatments (CK: standard Yoshida nutrient solution, CK-Salt: standard Yoshida nutrient solution + 0.6% (*w*/*w*) NaCl, Salt-OF: standard Yoshida nutrient solution + 0.6% (*w*/*w*) NaCl solution + 60-fold diluted fermented sheep manure solution).

Figure 1A shows the rice seedlings of the two rice cultivars, 19X (salt-sensitive) and HHZ (salt-tolerant), after 5 days under different treatment conditions. Under the CK treatment, both cultivars showed 100% survival rates, indicating that optimal performance was observed in both cultivars in the absence of salt stress. In the CK–salt treatment (0.6% (*w*/*w*) NaCl), the survival rate of 19X dropped significantly to approximately 6% (*p* < 0.05), while HHZ maintained a survival rate of 38%, highlighting the differential tolerance of the two cultivars to salt stress. HHZ exhibited higher survival rates compared with 19X under salt stress, demonstrating stronger adaptability to salt stress. Notably, the salt–OF treatment significantly improved survival rates for both cultivars compared with CK–salt (*p* < 0.05). The survival rate of 19X increased to around 25%, while HHZ achieved nearly 66%, comparable to its performance under CK–salt conditions. These findings underscore the critical role of OF in alleviating salt stress and enhancing survival rates, particularly for the salt-tolerant cultivar HHZ (Figure 1B).

The dynamic changes in the Normalized Difference Vegetation Index (NDVI) of the two rice cultivars, 19X and HHZ, under different treatments are presented in Figure 1C,D, reflecting the varietal differences in leaf health condition. Under the CK treatment, both cultivars maintained high NDVI values. This stability in NDVI during the tests indicated that both cultivars were in a healthy state under non-stress conditions. 19X exhibited a rapid decline in NDVI, from an initial value of approximately 0.56 to below 0.25 (*p* < 0.01), indicating significant suppression of photosynthesis by salt stress. In contrast, HHZ showed only a moderate decrease in NDVI on Day 2, maintaining values around 0.56, demonstrating greater tolerance to salt stress. The salt–OF treatment significantly increased the NDVI values of both cultivars compared with CK–salt (*p* < 0.01). For 19X, the decline in NDVI was observed after Day 2, while HHZ’s NDVI was maintained until Day 4. Moreover, HHZ consistently exhibited significantly higher NDVI values than 19X throughout the treatment period (*p* < 0.01).

The correlation between NDVI and the survival rate for the two rice cultivars under three treatment conditions (CK, CK–salt, and salt–OF) was examined. A strong positive correlation was observed, with a higher NDVI corresponding to an increased survival rate (R^2^ = 0.87, *p* < 0.01). This significant correlation highlights the predictive potential of NDVI for assessing seedling viability under salt stress (Figure 1E).

### 2.2. Metabolomic Profiling of Two Rice Cultivars Under Different Treatments

**Figure 2:**
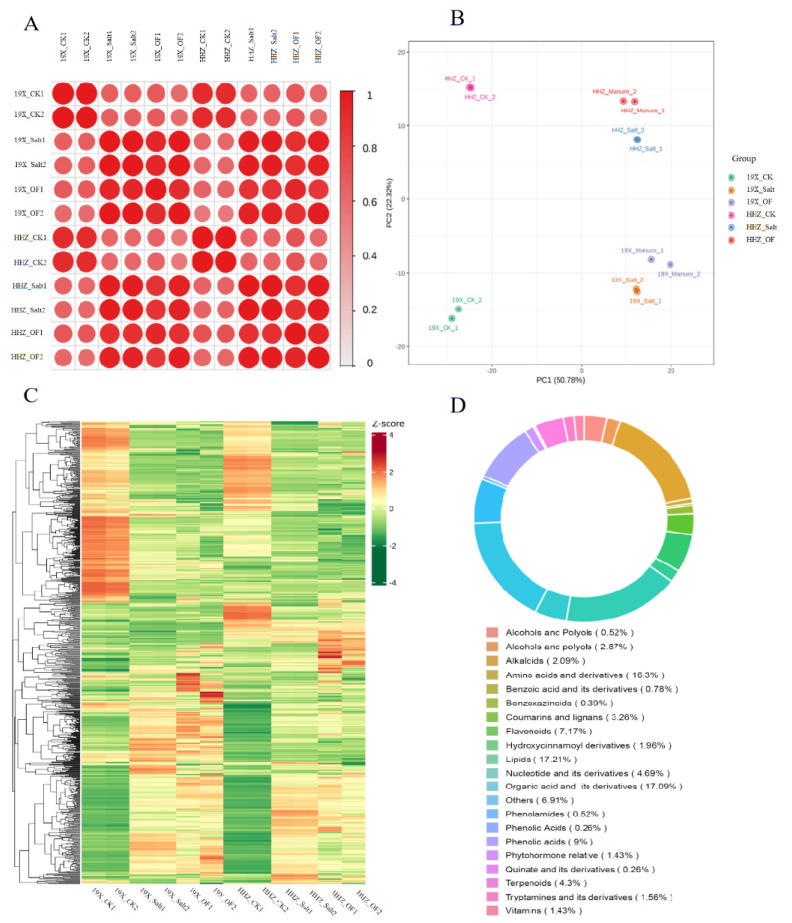
Metabolomic profiling of 767 metabolites in rice seedlings of two rice (*Oryza sativa* L.) cultivars (19X and HHZ) under three treatments (CK: standard Yoshida nutrient solution, Salt: standard Yoshida nutrient solution + 0.6% (*w*/*w*) NaCl, OF: standard Yoshida nutrient solution + 0.6% (*w*/*w*) NaCl solution + 60-fold diluted fermented sheep manure solution); correlation matrix using Pearson correlation coefficient (**A**); principal component analysis of the relative metabolites identified from six treatments (**B**); clustering heatmap of the metabolites identified from six treatments, hierarchical cluster analysis was used for metabolite clustering (**C**); classification of metabolites (**D**).

The correlation coefficients of metabolite abundance from two rice cultivars (HHZ and 19X) under three treatments: control (CK), salt stress, and salt stress with organic fertilizer are shown in Figure 2A. High internal correlations between replications indicate consistency in metabolic responses. Low cross-group correlation coefficients show distinct metabolic changes across treatments and between cultivars. The OF treatment increased similarity between the two cultivars.

The PCA plot highlights the clustering of metabolites of rice seedlings from the two cultivars under the three treatments (Figure 2B). The first two principal components (PC1 and PC2) explain 50.78% and 22.25% of the total variance, respectively. Samples from different replications were clustered closely, while distinct separation was observed between treatments and cultivars. The CK and salt-stress groups were well separated by PC1, and the two cultivars were separated by PC2. Meanwhile, the OF group exhibited closer clustering between the two cultivars, suggesting a unifying effect of OF on metabolic profiles under salt stress.

The heatmap visualizes the relative abundance (z-score) of 767 metabolites across all samples from two cultivars under three treatments (Figure 2C). Clear metabolic differences were observed between cultivars and treatments, with the salt treatment inducing widespread changes. The addition of OF appeared to moderate differences, reducing the metabolic divergence between HHZ and 19X under salt stress.

All 767 identified metabolites were classified among 20 categories (Figure 2D). Major categories included amino acids and their derivatives (16.33%), organic acids and their derivatives (17.09%), lipids (17.21%), flavonoids (7.17%), and nucleotides and their derivatives (4.69%). Minor categories included terpenoids (4.33%), vitamins (1.43%), and alkaloids (2.09%). The classification highlights the diversity of metabolic pathways involved in the rice stress response and the potential roles of specific compound classes in mediating resilience to salt stress under OF treatment.

### 2.3. Salt Tolerance Differences Between Two Rice Cultivars

**Figure 3:**
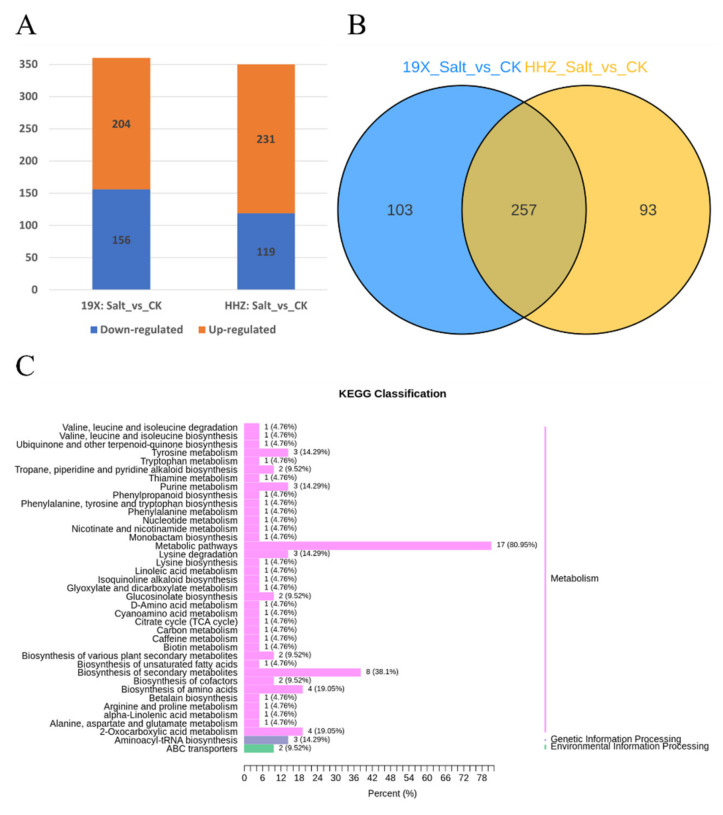
Differential analysis of salt tolerance between 19X and HHZ. (**A**) DEMs found in 19X and HHZ under salt stress compared with CK. (**B**) Venn diagram of metabolites distribution in 19X and HHZ under salt stress compared with CK. (**C**) KEGG pathways related to the 93 DEMs.

To analyze the differences in salt tolerance between 19X and HHZ, we conducted differential expression analyses between the salt treatment group and the control group (CK) for both cultivars. In 19X, a total of 360 differentially expressed metabolites (DEMs) were identified, including 204 upregulated and 156 downregulated metabolites (Figure 3A). For HHZ, 350 DEMs were identified, with 231 upregulated and 119 downregulated. Additionally, a comparison of the DEMs between the two cultivars revealed 257 common DEMs, while 93 DEMs were specifically differentially expressed in HHZ and 103 in 19X (Figure 3B). These results suggest that the 93 HHZ-specific DEMs contributed to the differences in salt tolerance between the two cultivars. Therefore, these 93 DEMs in HHZ were preliminarily identified as salt tolerance-related metabolites. These metabolites primarily included lipids, amino acids, organic acids, and phenolic acids (Figure S1) and were mainly distributed in metabolic pathways (Figure 3C).

**Figure 4:**
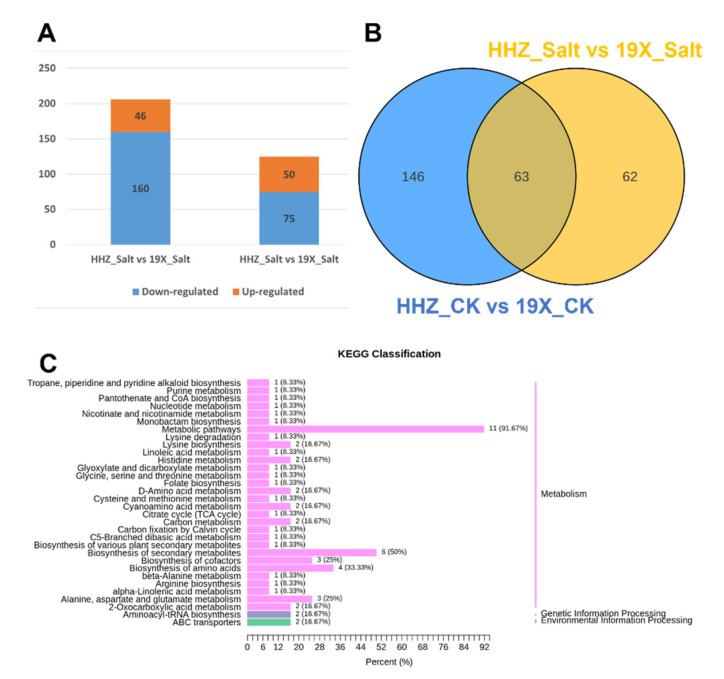
Differential analysis of salt tolerance between salt treatment and CK groups. (**A**) DEMs between HHZ and 19X under salt treatment and CK. (**B**) Venn diagram of DEMs distribution in salt treatment and CK groups. (**C**) KEGG pathways related to the 62 DEMs.

In addition, we performed differential analysis between the CK groups of the two cultivars (HHZ_CK vs. 19X_CK) and their salt-treated groups (HHZ_Salt vs. 19X_Salt). In the HHZ_CK vs. 19X_CK comparison, a total of 209 DEMs were identified, including 49 upregulated and 160 downregulated metabolites. In the HHZ_Salt vs. 19X_Salt comparison, 125 DEMs were identified, with 50 upregulated and 75 downregulated (Figure 4A). Among these, 63 DEMs were shared between the two comparison groups (Figure 4B). Notably, 62 DEMs were exclusively present in the HHZ_Salt vs. 19X_Salt comparison, which are highly likely to be associated with salt tolerance in rice. These DEMs included alcohols and polyols (3), alkaloids (2), amino acids and derivatives (16), benzoxazinoids (1), catechin and derivatives (1), coumarins and lignans (2), flavonoids (4), lipids (8), nucleotides and their derivatives (2), organic acids and their derivatives (10), phenolic acids (7), terpenoids (1), and others (8) (Figure S2). These 63 DEMs were annotated according to their metabolic pathways (Figure 4C). Further analysis revealed 12 common differentially expressed metabolites (DEMs) among the 93 DEMs identified in Figure 3 and the 62 DEMs in Figure 4 (Table 1). These shared DEMs included lipids, organic acids, and their derivatives, as well as phenolic acids. We selected these two analytical approaches to investigate specific physiological and biochemical responses to salt stress while minimizing confounding effects arising from the inherent vulnerabilities of salt-sensitive cultivars. It was expected that identifying responsive metabolites through these two methods would better highlight those associated with salt tolerance. This combined approach enhances our understanding of the survival mechanisms of rice under salt stress, which is essential for developing strategies to improve salt tolerance in rice cultivars.

### 2.4. Effects of OF on Salt Tolerance in 19X and HHZ

**Figure 5:**
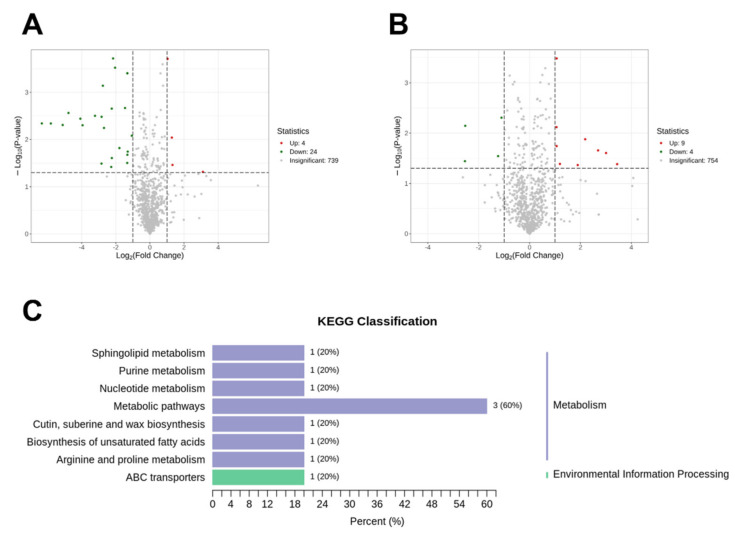
Differential metabolites and KEGG pathway analysis in HHZ and 19X under OF and salt treatment. (**A**) DEMs found in OF and salt groups in HHZ. (**B**) DEMs found in OF and salt groups in 19X. (**C**) KEGG pathways related with the 10 annotated metabolites out of 28 DEMs found in HHZ between OF and salt groups.

To investigate the effects of organic fertilizer on the salt tolerance of HHZ and 19X, we conducted comparative analyses of HHZ_ OF vs. HHZ_Salt and 19X_ OF vs. 19X_Salt. In HHZ, a total of 28 differentially expressed metabolites (DEMs) were identified, including 4 upregulated and 24 downregulated metabolites (Figure 5A, Table S1). In 19X, nine upregulated and four downregulated metabolites were observed (Figure 5B, Table S2). No overlapping DEMs were found between the two groups, indicating that the regulatory mechanisms of the organic fertilizer differed between the rice cultivars with varying salt sensitivity. We presume that the 28 DEMs identified in the salt-tolerant HHZ play a critical role in enhancing its salt tolerance. KEGG pathway analysis revealed that these DEMs are primarily associated with metabolism-related pathways (Figure 5C).

### 2.5. Correlation Analysis Between NDVI and Key Differential Metabolites

To assess the relationship between NDVI changes and key differential metabolites, correlation analysis was conducted across all treatments and both rice cultivars (Table S3). Among 53 differential metabolites identified, 26 of them were significantly correlated with NDVI, indicating their strong association with salt stress response and organic fertilizer regulation.

Positively correlated metabolites included tridecylic acid (R = 0.65, *p* = 0.019), cyclopentadecanolide (R = 0.62, *p* = 0.031), citric acid (R = 0.59, *p* = 0.042), pipecolic acid (R = 0.62, *p* = 0.030), ceramide (d18:1/16:0) (R = 0.68, *p* = 0.01), and astilbin (R = 0.63, *p* = 0.028), suggesting their potential roles in maintaining plant health and stress resilience. Notably, pindolol (R = 0.84, *p* = 0.0005) showed the strongest positive correlation with NDVI, highlighting its significant contribution to stress mitigation.

In contrast, octadecapenten-3-one (R = −0.65, *p* = 0.019), inosine (R = −0.52, ns), and HIAA (R = −0.90, *p* = 0.001) were negatively correlated with NDVI, suggesting their association with stress-induced metabolic disruptions. Additionally, N2-dimethylguanosine (R = −0.61, *p* = 0.035) exhibited a negative correlation, indicating potential trade-offs between nucleic acid modifications and plant stress responses.

These findings indicate that NDVI changes corresponded with metabolic shifts, supporting the role of NDVI as an effective indicator of physiological responses to salt stress and organic fertilizer application.

## 3. Discussion

### 3.1. Effects of Salt Stress on Different Rice Cultivars

In this study, the physiological responses to salt stress in two rice cultivars, the salt-sensitive 19X and the salt-tolerant HHZ, were investigated, and significant differences in their reactions were identified. Salt stress was found to affect plants by inducing osmotic stress, which disrupted water absorption and increased ion toxicity, therefore resulting in symptoms such as chlorosis, restricted root development, and growth inhibition [22]. As reported by Munns and Tester [3], salt stress interferes with plant water balance and ion assimilation, adversely impacting overall growth. In this study, 19X was observed to experience pronounced inhibition of growth under salt stress. Its normalized difference vegetation index (NDVI) values were found to decrease sharply within two days, reflecting a reduction in photosynthetic efficiency and survival rates. In contrast, HHZ was observed to maintain relatively high NDVI values under the same conditions, demonstrating robust growth vitality in response to salt stress.

Significant salt tolerance in HHZ was revealed in this study, as its survival rates and NDVI values remained at high levels under salt stress. This phenomenon was attributed to HHZ’s more efficient ion regulation mechanisms and stronger antioxidant systems. As noted by Balasubramaniam et al. [23], salt-tolerant crops have generally been found to employ specific physiological mechanisms to mitigate salt-induced damage. These mechanisms include enhanced root water uptake, increased accumulation of osmolytes, and reduced oxidative stress through robust antioxidant enzyme systems. Through these mechanisms, HHZ was able to maintain better hydration and lower ion toxicity under salt stress. Conversely, 19X, being a salt-sensitive variety, was observed to exhibit significant metabolic disorders under salt stress, resulting in sharp declines in growth and photosynthetic efficiency. As well as these differences in growth metrics and NDVI data, variations in metabolic products were also recorded. The NDVI of 19X was found to decrease dramatically in the early stages of salt stress (Figure 1B), reflecting its physiological vulnerability to salinity.

The effectiveness of NDVI as a non-invasive, real-time monitoring tool was further validated in this study. NDVI, which reflects chlorophyll content and photosynthetic efficiency, was demonstrated to be sensitive to changes in photosynthetic capacity during the early stages of salt stress [24]. As a result, variations in NDVI were shown to serve as crucial indicators of early stress responses in plants. Under salt stress, the rate of NDVI decline was found to correlate directly with the physiological stress experienced by the plant.

### 3.2. The Role of Organic Fertilizers in Mitigating Salt Stress

This study demonstrated that fermented sheep manure applied as an organic fertilizer exhibited significant regulatory effects on rice growth and metabolism under salt stress, effectively improving the survival rates of different rice cultivars. Under salt stress, plants were reported to activate a series of metabolic response mechanisms, including the accumulation of osmoprotectants (such as proline and glycerol), the enhancement of antioxidant enzyme activities, and the regulation of secondary metabolite synthesis [25]. Organic fertilizers were shown to improve plant nutritional status, promote metabolic activity, and enhance adaptation to salt stress.

In this study, significant metabolic changes were observed in both 19X and HHZ after the addition of organic fertilizer, particularly in key metabolites such as amino acids, lipids, and organic acids (Figure 5). Amino acids, such as proline, have been confirmed to be critical osmotic regulators in plants responding to salt stress [25]. The synthesis of these osmoprotectants was found to be more efficient under the influence of organic fertilizers, alleviating osmotic stress caused by salinity. Additionally, antioxidant enzyme systems, including catalase (CAT) and superoxide dismutase (SOD), have been shown to exhibit enhanced activity under organic fertilizer treatment, helping to reduce oxidative stress induced by salt stress and mitigate the damage caused by reactive oxygen species (ROS) to plant cells [26].

HHZ was found to exhibit stronger salt tolerance, potentially attributed to its more effective regulation of salt absorption and compartmentalization in roots [27,28]. In contrast, 19X showed improvements with the application of organic fertilizer but still could not respond to salt stress as effectively as HHZ. Metabolomic analysis revealed that under salt stress, HHZ treated with organic fertilizer involved a greater number of metabolites in antioxidant responses and osmotic regulation, whereas the metabolic responses in 19X were relatively weaker. These findings suggest that salt-tolerant cultivars might possess stronger endogenous regulatory capabilities, which could be further enhanced by the application of organic fertilizers to achieve more effective salt-resistance mechanisms.

### 3.3. Effects of Salt Stress and Organic Fertilizer on Metabolites

Salt stress has been found to induce changes in the internal environment of plants, altering their metabolic pathways. Sodium (Na^+^) and chloride (Cl^−^) ion accumulation was observed to disrupt ionic balance within plant cells [29]. High concentrations of Na^+^ were reported to compromise cell membrane stability, further affecting normal plant metabolism [30]. Additionally, salt stress has been shown to induce oxidative stress, as the accumulation of reactive oxygen species (ROS) causes damage to cell membranes, proteins, and DNA, thereby inhibiting normal plant growth and development [31]. To mitigate these adverse effects, plants have been observed to activate a series of metabolic responses, including increased synthesis of osmotic regulators, enhanced accumulation of antioxidants, modulation of lipid metabolism, and alterations in amino acid and carbohydrate metabolism [18]. These responses have been found to help plants maintain cellular hydration, reduce oxidative damage, and stabilize energy metabolism. In this study, salt stress was shown to induce large-scale changes in rice metabolites. Significant changes were observed in amino acids, lipids, and organic acids under salt treatment. Notably, the accumulation of amino acids such as proline and glutamic acid highlighted the activation of osmotic regulation and antioxidant mechanisms (Figure 3 and Figure 4). These metabolites have been found to contribute to maintaining cellular hydration and alleviating oxidative damage during salt stress.

There were 12 common differentially expressed metabolites (DEMs) among the 93 DEMs identified in Figure 3 and the 62 DEMs in Figure 4 (Table 1). Under normal conditions, no significant differences were detected between these 12 metabolites in the salt-tolerant variety HHZ and the salt-sensitive variety 19X. However, under salt stress, significant changes in these 12 metabolites were observed in HHZ. Not only were these changes found to be significant compared with HHZ under control conditions, but they were also markedly different from the response of 19X under salt stress. This suggested that these metabolites played important roles in the response to salt stress, probably closely related to HHZ’s salt tolerance.

In detail, these substances were found to be closely linked through metabolic pathways, collectively forming a complex metabolic network enabling plants to respond to salt stress. Citric acid is a key intermediate in the TCA cycle, providing cellular energy and serving as a carbon skeleton for synthesizing amino acids (e.g., pipecolic acid) and fatty acids (e.g., myristoleic acid, tridecylic acid) [32]. Recent studies highlight its role in enhancing plant salt tolerance by regulating antioxidant defenses, reducing ROS accumulation, promoting osmotic adjustment substances, and improving growth and physiology. Tahjib-Ul-Arif et al. [33] reviewed its mechanisms in abiotic stress responses, while Ali et al. [34] showed that exogenous citric acid enhanced the growth of eggplant under salt stress. Chakrobortty et al. [35] found that seed priming and citric acid application improved okra germination and seedling growth under salt stress. These findings support the role of citric acid as an eco-friendly indicator of stress tolerance in rice plants. In addition to citric acid, several candidate compounds selected in this study have been shown in previous research to be closely associated with plant responses to salt stress. For example, HTPA has been reported to exhibit upregulated expression under salt stress [36]. Pipecolic acid is known to accumulate significantly during salt stress [37,38], and myristoleic acid levels are markedly increased under salt stress [39]. Maleamic acid has been identified as a contributory compound related to salt stress responses [40]. Additionally, other selected candidate compounds in this study, such as tridecylic acid, propentofylline, octadeca penten-3-one, 14,16-dihydroxy-benzoxacyclotetradecine-dione, and cyclopentadecanolide, have not yet been reported in the context of salt stress. These 12 compounds hold potential as candidate biomarkers for the selection of salt-tolerant varieties, though their specific roles and mechanisms in salt stress responses require further investigation in future studies.

The application of organic fertilizer significantly altered the metabolic profiles of the rice. Under salt stress conditions, organic fertilizer was found to promote the synthesis of key metabolites such as amino acids, lipids, and organic acids (Figure 5C). These changes were observed to enhance the activity of antioxidant enzymes, effectively reducing the accumulation of ROS and protecting cells from oxidative stress [41]. Additionally, the significant regulation of lipid metabolism by organic fertilizer was shown to further support the plants’ responses to salt stress (Figure 5C). Lipids, which are critical components of cellular membranes, have been found to play roles in signal transduction and energy storage [42]. Salt stress has been reported to disrupt lipid metabolism, damaging membrane structures and impairing cellular function. However, organic fertilizer can improve plant nutritional status, promote lipid synthesis and repair, maintain membrane stability, and facilitate adaptation to salt stress. Furthermore, organic fertilizer can enhance nutrient uptake and root growth, regulate metabolic pathways, and increase antioxidant capacity, thereby mitigating oxidative damage caused by salt stress [16]. Organic fertilizer has been shown to provide important support for alleviating salt stress through metabolic regulation and maintaining plant physiological stability.

Metabolomic analysis further revealed the interaction between salt stress and organic fertilizer at the metabolite level. Significant changes in rice metabolites were identified in the salt treatment group (Figure 3 and Figure 4), while the impact of salt stress on metabolites was notably alleviated in the organic fertilizer treatment group (Figure 5). Particularly, distinct trends were observed in the changes in amino acids and lipid metabolites between the salt treatment and organic fertilizer treatment groups. In terms of amino acids, salt stress induced the accumulation of osmotic regulators such as proline and glutamic acid, which contribute to cellular hydration and osmotic balance. In the organic fertilizer treatment group, the accumulation of these amino acids was further enhanced, suggesting that the organic fertilizer not only facilitated the synthesis of osmotic regulators but also improved the plants’ nutritional status, thereby boosting salt stress tolerance [43]. Moreover, changes in lipid metabolites highlight the role of organic fertilizer in maintaining membrane integrity and enhancing antioxidant capacity.

The effects of organic fertilizer were found to vary between the rice cultivars. In the salt-tolerant cultivar HHZ (Table S1), the application of organic fertilizer reduced the rice’s dependence on osmotic regulatory substances (alcohols and organic acids were downregulated), suggesting an improvement in water supply and salt ion balance and thereby minimizing the need for excessive accumulation of these compounds [44,45]. It also decreased lipid metabolites associated with membrane stability and cell death (DiHOME, Ceramide), indicating enhanced membrane integrity, reduced oxidative damage, and lower levels of programmed cell death [46]. Additionally, the upregulation of inosine promoted energy metabolism, potentially improving the rice’s ability to recover from salt stress [47]. The reduced presence of the auxin degradation product HIAA suggests increased auxin metabolism efficiency, which may enhance rice growth and root development under salt stress [48]. Overall, organic fertilizer mitigated the intensity of stress responses, bringing the metabolic state of rice closer to normal growth conditions. In salt-sensitive cultivar 19X (Table S2), the application of organic fertilizer enhanced the antioxidant capacity of the rice, as evidenced by the upregulation of flavonoids (astilbin) and alkaloids (1,2,3,4-tetrahydro-β-carboline-3-carboxylic acid), which may have increased antioxidant enzyme activity and reduced oxidative damage caused by salt stress [49]. Additionally, the upregulation of propionylcarnitine and phospholipid metabolites suggests an improvement in fatty acid metabolism, enabling better energy utilization and enhancing salt tolerance [50]. The upregulation of propionylcarnitine, a key metabolite involved in fatty acid transport and mitochondrial energy production, suggests that organic fertilizer may have promoted efficient energy metabolism, supporting plant resilience under salt stress [51]. In conclusion, in the salt-sensitive rice cultivar 19X, organic fertilizer improved salt stress tolerance by enhancing antioxidant capacity, optimizing energy metabolism, reducing stress-related signals, and regulating gene expression.

The metabolic regulation mechanisms by which organic fertilizer alleviates salt stress differ significantly between the salt-sensitive rice cultivar 19X and the salt-tolerant cultivar HHZ, reflecting their intrinsic salt tolerance capabilities. In 19X, organic fertilizer primarily mitigates oxidative stress by enhancing antioxidant defenses, as evidenced by the upregulation of flavonoids (astilbin) and alkaloids (1,2,3,4-tetrahydro-β-carboline-3-carboxylic acid), helping to reduce the accumulation of reactive oxygen species (ROS). Additionally, organic fertilizer improves energy metabolism by increasing propionylcarnitine and phospholipid metabolites, thereby enhancing fatty acid metabolism and ensuring adequate energy supply under stress conditions. In contrast, HHZ exhibits inherent salt tolerance and a more stable antioxidant and osmotic regulation system. The primary role of organic fertilizer in HHZ is to optimize membrane stability and ion homeostasis, as indicated by the downregulation of lipid peroxidation products such as DiHOME and ceramides, and this helps to reduce membrane damage. Unlike 19X, which relies on fatty acid metabolism for energy, HHZ predominantly utilizes nucleotide metabolism (e.g., upregulation of inosine) to maintain energy supply and enhance recovery from salt stress. These metabolic differences suggest that organic fertilizer plays a broad regulatory role in 19X, compensating for its weaker salt tolerance, whereas in HHZ, it fine-tunes existing adaptive mechanisms. This finding underscores the necessity of developing cultivar-specific fertilization strategies based on distinct metabolic responses, optimizing stress resilience and improving crop performance under saline conditions.

### 3.4. Integrating NDVI and Metabolomics for Assessing Rice Responses to Salt Stress

The Normalized Difference Vegetation Index (NDVI) is a non-invasive spectral index that is widely used for monitoring plant health and stress responses by capturing spectral reflectance from leaves [52]. NDVI provides a rapid evaluation of photosynthetic efficiency and growth status, making it a valuable tool for assessing early physiological changes under stress [53,54,55]. In this study, the NDVI values of the salt-sensitive rice variety 19X declined rapidly under salt stress, indicating severe photosynthetic impairment, while the salt-tolerant variety HHZ maintained higher NDVI values, demonstrating better adaptability.

Metabolomics is a high-throughput analytical technique that enables detailed molecular insights into plant stress adaptation by detecting metabolite shifts [56,57]. Our results showed that salt stress significantly altered the rice plants’ metabolism, particularly in amino acid and lipid pathways. HHZ exhibited superior metabolic regulation, with the accumulation of proline and antioxidants suggesting a stronger biochemical defense against salt stress. Significant changes in spectral reflectance often correspond with metabolite accumulation [53,58], linking external phenotypic traits to internal molecular responses.

To further investigate this relationship, we analyzed the correlation between NDVI and key differential metabolites under different treatments. The results confirmed that NDVI changes were significantly correlated with multiple stress-related metabolites, indicating its reliability as a physiological indicator of metabolic shifts [59].

Traditional screening methods for salt tolerance have relied on growth analysis and scoring, an approach that lacks precision in capturing dynamic stress responses [59]. Integrating NDVI with metabolomics enhances screening efficiency by connecting early phenotypic changes with molecular mechanisms. This approach facilitates early identification of salt stress responses, enabling precise screening of salt-tolerant cultivars with strong metabolic regulatory capacities.

## 4. Materials and Methods

### 4.1. Experimental Materials

This study utilized two rice cultivars: Huanghuazhan (HHZ) (salt-tolerant) and 19Xiang (19X) (salt-sensitive), which are both typical indica rice varieties widely cultivated in southern China. The experiment consisted of three treatments: control (CK), 0.6% (*w*/*w*) salinity stress (NaCl), and 0.6% (*w*/*w*) salinity stress with organic fertilizer (NaCl + OF). The cultivation was performed using hydroponic systems with four replicates per treatment.

### 4.2. Experimental Design

For cultivation and preparation, rice seedlings were grown in hydroponic boxes in a walk-in growth chamber at a constant temperature of 30 °C and a relative humidity of 70%. For each cultivar, 96 fully developed seeds were sown per replicate. After surface sterilization, the seeds were placed on germination substrate on 96-well plates with the cone tips removed. Upon emergence of the seedlings, they were transferred to hydroponic boxes and grown in water for three days. Subsequently, the water was replaced with nutrient solution. To ensure uniform growth, seedlings were cultured in nutrient solution for one week at the three-leaf stage, and inconsistent individuals were removed, leaving 60 uniform seedlings per replicate. All plants were pre-cultured in a standard Yoshida nutrient solution.

For the salinity stress treatment, salinity stress was applied by adding 0.6% (*w*/*w*) NaCl to the nutrient solution. For the salinity and OF treatment, OF derived from 60-fold diluted fermented sheep manure solution was added to the saline nutrient solution. The treatment solutions were renewed every two days to maintain consistent concentrations of salt and organic fertilizer.

The fermentation process use a fermentation agent primarily composed of various probiotics and related enzymes, including *Bacillus*, *Saccharomyces* (yeast), photosynthetic bacteria, *Acetobacter* (acetic acid bacteria), *Lactobacillus* (lactic acid bacteria), *Actinomycetes*, and *Trichoderma*. It was sourced from Shandong Junde Biotechnology Co., Ltd (Economic Development Zone, Zhucheng City, Weifang, Shandong Province, China).

A total of 300 g of sheep manure, 100 g of brown sugar, and 2000 mL of purified water was measured and poured into an 8 L fermentation bucket. Then, 0.6 g of the aforementioned fermentation agent was added. The mixture was stirred evenly and placed in a 30 °C incubator for fermentation. To ensure uniformity, the mixture was stirred for 5 min every 12 h, and its pH value and electrical conductivity (EC) were measured. To maintain fermentation stability across batches, environmental conditions were strictly controlled, and the pH value and EC were continuously monitored throughout the fermentation process. When these two parameters remained unchanged for three consecutive measurements, indicating stabilization, the fermentation product was filtered. The filtrate was sterilized using a UV lamp for 30 min and then stored in a refrigerator at 4 °C. The residue was placed in a 30 °C drying oven for low-temperature drying, after which it was sealed and stored.

Two replicates were designated for NDVI measurement and metabolomic sampling. The other two replicates were returned to standard nutrient solution after five days of treatment to observe survival rates after one week of recovery. The criterion for determining survival rate was the presence of newly grown green leaves during the recovery cultivation period.

### 4.3. Measurement Indicators

#### 4.3.1. NDVI Measurements

NDVI was measured daily for five days following the onset of treatments, using a handheld Polypen PSI spectrometer (Drasov 470, 66424 Drasov, Czech Republic). NDVI measurements were consistently taken from the center of penultimate leaves of the 6 marked plants in each plot. This selection ensured uniformity across measurements and reduced variation caused by differences in leaf age or exposure. Measurements were conducted at the same time each afternoon to minimize fluctuations due to the plants’ circadian rhythm. Additionally, all measurements were performed in the walk-in growth chamber to avoid variability caused by environmental changes. NDVI was calculated as follows:

NDVI = (NIR − R)/(NIR + R)

where NIR represents near-infrared reflectance and R denotes red-light reflectance. The NDVI data were used to analyze dynamic changes in photosynthetic performance under different treatments.

#### 4.3.2. Plant Sampling and Metabolomic Analysis

On the fifth day of treatment, ten plants were randomly selected from each replicate for sampling. Samples were immediately frozen in liquid nitrogen and stored at −80 °C for metabolomic analysis. Non-targeted metabolomic profiling was performed using a high-resolution liquid chromatography–mass spectrometry (LC-MS) system whith Thermo Scientific Q Exactive, (Thermo Fisher Scientific Inc. 168 Third Avenue, Waltham, MA 02451, USA). The procedure is described as follows.

For preparation or samples, tissues were homogenized and extracted using methanol–water (7:3, *v*/*v*) extraction solution. Extracts were subjected to ultrasonic treatment and centrifugation. Extracts were separated via liquid chromatography and analyzed using mass spectrometry under positive and negative ionization modes. The scanning mass ranged from *m*/*z* 100 to 1000 with an accumulation time of 0.10 s. The scanning mode was full MS/ddMS2. The recorded data were processed using Compound Discoverer (CD) 3.3 software to obtain the mass-to-charge ratio (*m*/*z*), retention time, and MS/MS2 fragmentation information of all the detected compounds. To ensure the reliability and reproducibility of the data, quality control samples were prepared by pooling equal volumes from all biological samples and analyzed at regular intervals throughout the run to monitor instrument stability. Lidocaine was used as an internal standard to confirm baseline instrument stability. In addition, mass drift correction and alignment of retention times were performed to ensure comparability across samples. For data processing, peak area normalization was applied for signal normalization. Batch effect correction was not necessary as all samples were analyzed in a single batch. Identification of metabolites was conducted using both primary MS (*m*/*z* ratio) and secondary MS/MS fragmentation spectra, with spectral matching against multiple databases, including KEGG, HMDB, and METLIN. Identified metabolites were classified according to the Metabolomics Standards Initiative (MSI) guidelines, with Level 1 metabolites confirmed by spectral matching with authentic standards and Level 2 metabolites identified based on high-confidence spectral similarity.

Principal component analysis (PCA) and partial least squares discriminant analysis (PLS-DA) were employed to identify significantly different metabolites (VIP > 1, *p* < 0.05). KEGG pathway enrichment analysis was performed to investigate the role of these metabolites in salinity stress response.

#### 4.3.3. Survival Rate Observation

For the remaining two replicates, plants were returned to standard nutrient solution after five days of treatment and maintained for one additional week. After recovery, the plants were observed in order to determine whether new leaves had emerged. Plants with new leaf growth were considered survivors, while those without new leaf growth were classified as dead. The survival rate was calculated as the ratio of surviving plants to the initial 60 plants per replicate, providing insights into the combined effects of salinity stress and organic fertilizer on plant resilience.

#### 4.3.4. Data Analysis

All statistical analyses were conducted using SPSS 26.0. Dynamic NDVI changes were compared across treatments using ANOVA, with significance set at *p* < 0.05 or *p* < 0.01. Metabolomics data were analyzed using R software (https://cran.r-project.org/) with the “MetaboanalystR” package for visualization and pathway enrichment. The differentially expression metabolites (DEMs) were determined by absolute log_2_ Fold Change (|log_2_Fold Change| ≥ 1) and *p*-value < 0.05, *p*-value was calculated by Student’s *t* test. Survival rates were analyzed using one-way ANOVA, with the significance level also set at *p* < 0.05. Principal component analysis (PCA) was performed using the “prcomp function” in R, with z-score normalization prior to PCA. Pearson correlation coefficients (PCCs) were calculated to represent the correlations between two samples or metabolites. Hierarchical cluster analysis (HCA) was employed to assess the clustering of metabolites in the clustering heatmap.

## 5. Conclusions

This study demonstrated that organic fertilizer significantly enhanced rice tolerance to salt stress through distinct metabolic mechanisms, as revealed by integrating analysis of NDVI and metabolomics. The findings indicated that organic fertilizer improved survival rates and physiological responses in both salt-sensitive and salt-tolerant rice cultivars, albeit through different regulatory pathways. The identification of key metabolites and their correlation with NDVI highlights the potential of combining spectral phenotyping with metabolomics analysis for precise evaluation of salt tolerance. These insights contribute to a deeper understanding of the metabolic basis of salt tolerance and emphasize the importance of targeted fertilization strategies to optimize stress resilience in rice.

Beyond confirming the beneficial effects of organic fertilizer, this study underscores the necessity of cultivar-specific fertilization approaches, given the distinct metabolic responses observed in different rice varieties. The strong correlation between NDVI and key metabolic markers suggests that NDVI can be a powerful tool for real-time monitoring of salt stress responses in rice. However, the composition of organic fertilizer may vary depending on raw materials and fermentation conditions, and the effects of organic fertilizer may vary under different culture conditions or soil types. Future research should focus on validating the functional roles of the newly identified metabolites revealed in the current study, exploring the interactions between organic fertilizer components and plant metabolic pathways, and optimizing fertilization strategies tailored to different genotypes under different culture conditions. These findings offer valuable applications for sustainable agriculture, precision farming, and the development of salt-tolerant rice varieties.

## Figures and Tables

**Table 1:** Intersection of 93 DEMs from Figure 3 and 62 DEMs from Figure 4.

Substance Name	Class	HHZ_Salt_vs._CK	HHZ_Salt_vs._19X_Salt
(±) 13-HpODE	Lipids	up	up
(±) 8,9-DiHETE	Lipids	down	down
14,16-dihydroxy-3-methyl-3,4,5,6,7,8,9,10-octahydro-1H-2-benzoxacyclotetradecine-1,7-dione	Benzoxazinoids	up	up
Citric acid	Organic acids and derivatives	down	down
Cyclopentadecanolide	Lipids	down	down
(2S,4S)-4-hydroxy-2,3,4,5-tetrahydrodipicolinic acid (HTPA)	Amino acids and derivatives	down	down
Maleamic acid	Organic acids and derivatives	down	down
Myristoleic acid	Lipids	down	down
Octadeca penten-3-one	Lipids	up	up
Pipecolic acid	Amino acids and derivatives	down	down
Propentofylline	Others	down	down
Tridecylic acid	Organic acids and derivatives	down	down

## Data Availability

The original contributions presented in this study are included in the article; further inquiries can be directed to the corresponding author.

## Supplementary Materials

The following supporting information can be downloaded at https://www.mdpi.com/article/10.3390/plants14060902/s1: Figure S1: Specific metabolites classified in response to salt stress in HHZ compared with 19X; Figure S2. Specific metabolites classified in salt-tolerant cultivar HHZ; Table S1. Differential metabolites identified in the salt-tolerant rice cultivar HHZ in response to organic manure under saline stress; Table S2. Differential metabolites identified in the salt-sensitive rice variety 19x in response to organic manure under saline stress. Table S3. Correlation analysis between NDVI and key differential metabolites in response to salt stress and organic fertilizer under different treatments in two rice cultivars.
