# Supplementary material for: Unveiling the Impact of Organic Fertilizer on Rice (Oryza sativa L.) Salinity Tolerance: Insights from the Integration of NDVI and Metabolomics
Source: Plants (Basel). 2025 Mar 13;14(6):902. doi: 10.3390/plants14060902 (PMC11946821; doi:10.3390/plants14060902)
Supplement: Supplementary file 1 [file plants-14-00902-s001.zip › plants-3459962-supplementary.pdf]

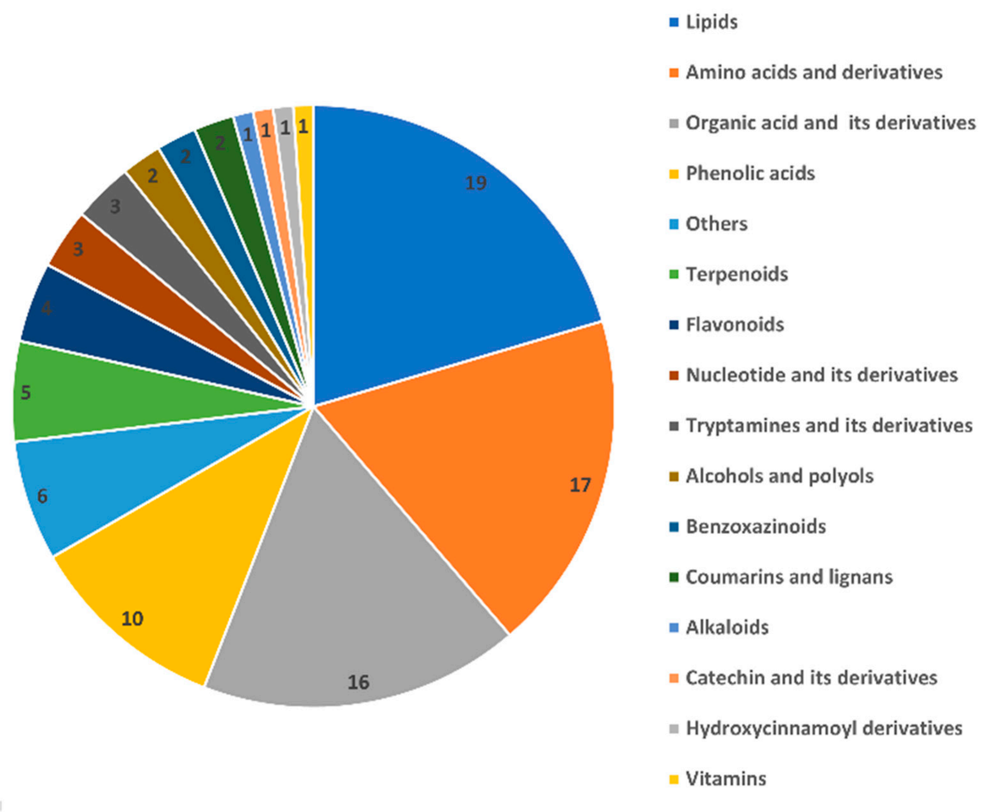

Figure S1. Specific metabolites classified in response to salt stress in HHZ compared to 19X.

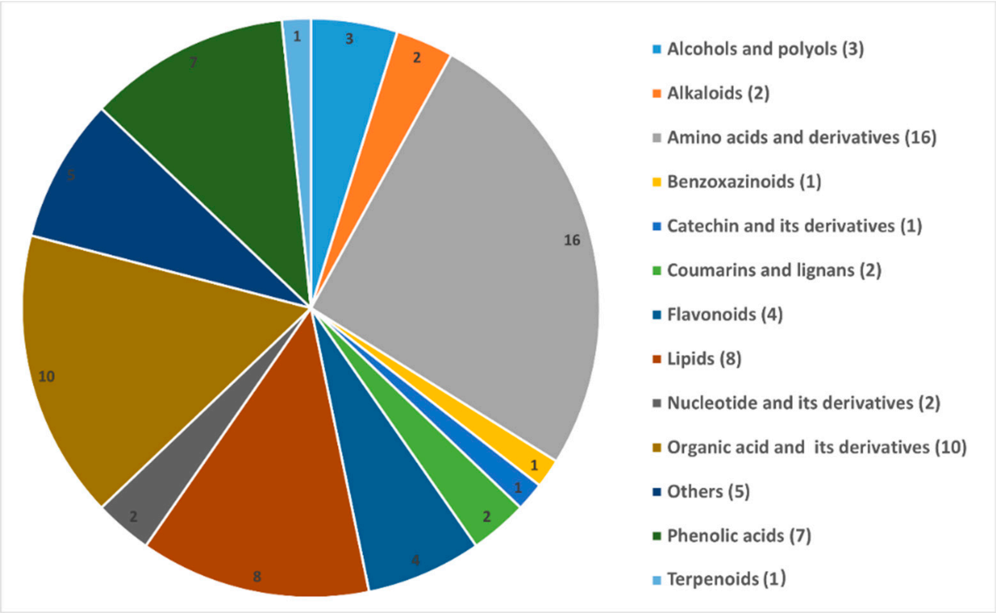

Figure S2. Specific metabolites classified in salt-tolerant cultivar HHZ.

Table S1. Differential metabolites identified in the salt-tolerant rice cultivar HHZ in response to organic manure under saline stress.

Substance Name	Class	HHZ_Manure_vs_Salt
Heptaethylene Glycol	Alcohols and polyols	down
Mandenol	Alcohols and polyols	down
HO-dPEG8-OH	Alcohols and polyols	down
DL-Lysine	Amino acids and derivatives	down
11-Aminoundecanoic acid	Amino acids and derivatives	down
13-Aminotridecanoic acid	Amino acids and derivatives	down
14,16-dihydroxy-3-methyl-3,4,5,6,7,8,9,10-octahydro-1H-2-benzoxacyclotetradecine-1,7-dione	Benzoxazinoids	down
(±)12(13)-DiHOME	Lipids	down
(±)9(10)-DiHOME	Lipids	down
8(S),15(S)-DiHETE	Lipids	down
(2R)-1-{[(2-Aminoethoxy)(hydroxy)phosphoryl]oxy}-3-hydroxy-2-propanyl (9Z)-9-tetradecenoate	Lipids	down
Erucic acid	Lipids	down
2,3-Dihydroxypropyl stearate	Lipids	up
Platelet-activating factor	Lipids	down
N,N-Bis(2-hydroxyethyl)dodecanamide	Lipids	down
C14-Dihydroceramide	Lipids	down
Ceramide (d18:1/16:0)	Lipids	down
C16-Dihydroceramide	Lipids	down
Inosine	Nucleotide and its derivatives	up
5-{(1R,2S,6R)-2,4-Dihydroxy-6-[(1E,3S)-3-hydroxy-1-octen-1-yl]cyclohexyl}pentanoic acid	Organic acid and its derivatives	down
2-Octenoic acid	Organic acid and its derivatives	down
3-Methylazelaic acid	Organic acid and its derivatives	down
9,10-Dihydroxystearic acid	Organic acid and its derivatives	down
4-acetamidobutyrate	Organic acid and its derivatives	up

(5E)-2,10-Diamino-5-{[(5-amino-5-carboxypentyl)amino]methyl}-5-undecenedioic acid (non-preferred name)	Organic acid and its derivatives	down
Pifithrin	Others	up
(1S,2S,6S,9S,10S,11R,12R,13S,14S,15S,16R,18S,19S,22S,23R,25R)-6,10,19-trimethyl-24-oxa-4-azaheptacyclo hexacosane-10,12,13,14,16,22,23-heptol	Terpenoids	down
HIAA	Tryptamines and its derivatives	down

Table S2. Differential metabolites identified in the salt-sensitive rice variety 19x in response to organic manure under saline stress.

**Substance Name**	**Class**	**19X_Manure_vs_Salt**
(2R)-3-({[(2S)-2,3-Dihydroxypropoxy](hydroxy)phosphoryl}oxy)-2-hydroxypropyl (9Z,12Z)-9,12-octadecadienoate	Lipids	up
(2E)-3-Phenyl-2-propen-1-yl 6-O-beta-D-arabinofuranosyl-beta-D-glucopyranoside	Flavonoids	up
cis-3-Hexenyl phenylacetate	Organic acid and its derivatives	up
(1R)-6-Hydroxy-7-methoxy-1-methyl-1,2,3,4-tetrahydro-1-isoquinoliniumcarboxylate	Alkaloids	down
1,2,3,4-Tetrahydro-Î²-carboline-3-carboxylic acid	Alkaloids	up
Pindolol	Organic acid and its derivatives	down
Astilbin	Flavonoids	up
N6,N6,N6-Trimethyl-L-lysine	Amino acids and derivatives	down
Piracetam	Nucleotide and its derivatives	up
caroxazone	Amino acids and derivatives	down
N2-Dimethylguanosine	Nucleotide and its derivatives	up
propionylcarnitine	Amino acids and derivatives	up
HHL	Organic acid and its derivatives	up

Table S3. Correlation analysis between NDVI and key differential metabolites in response to salt stress and organic fertilizer under different treatments in two rice cultivars.

Source	Name	R	P-Value
HHZ_Salt_vs_CK ∩ HHZ_Salt_vs_19X_Salt (12)	octadecapenten-3-one	-0.65	0.019
	(±)13-HpODE	-0.55	ns
	14,16-dihydroxy-3-methyl-3,4,5,6,7,8,9,10-octahydro-1H-2-benzoxacyclotetradecine-1,7-dione	-0.43	ns
	Maleamic acid	0.46	ns
	Myristoleic acid	0.57	0.048
	Citric acid	0.59	0.042
	Cyclopentadecanolide	0.62	0.031
	(±)8,9-DiHETE	0.62	0.031
	Pipecolic acid	0.62	0.030
	Tridecylic acid	0.65	0.019
	(2S,4S)-4-hydroxy-2,3,4,5-tetrahydrodipicolinic acid	0.75	0.005
	Propentofylline	0.79	0.002
HHZ_Manure_vs_Salt (28)	Heptaethylene Glycol	0.41	ns
	HIAA	-0.9	0.001
	Inosine	-0.52	ns
	14,16-dihydroxy-3-methyl-3,4,5,6,7,8,9,10-octahydro-1H-2-benzoxacyclotetradecine-1,7-dione	-0.43	ns
	Platelet-activating factor	-0.24	ns
	4-acetamidobutyrate	-0.14	ns
	DL-Lysine	0.04	ns
	2-Octenoic acid	0.18	ns
	3-Methylazelaic acid	0.18	ns
	N,N-Bis(2-hydroxyethyl)dodecanamide	0.26	ns
	HO-dPEG8-OH	0.34	ns
	unknown	0.45	ns
	Erucic acid	0.47	ns
	unknown	0.48	ns
	11-Aminoundecanoic acid	0.55	ns
	(5E)-2,10-Diamino-5-{[(5-amino-5-carboxypentyl)amino]methyl}-5-undecenedioic acid (non-preferred name)	0.58	0.04
	Mandenol	0.59	0.04
	(±)12(13)-DiHOME	0.61	0.03
	(±)9(10)-DiHOME	0.61	0.03

	13-Aminotridecanoic acid	0.62	0.03
	2,3-Dihydroxypropyl stearate	0.67	0.02
	9,10-Dihydroxystearic acid	0.67	0.02
	Ceramide (d18:1/16:0)	0.68	0.01
	5-{(1R,2S,6R)-2,4-Dihydroxy-6-[(1E,3S)-3-hydroxy-1-octen-1-yl]cyclohexyl}pentanoic acid	0.68	0.01
	8(S),15(S)-DiHETE	0.69	0.01
	C14-Dihydroceramide	0.69	0.01
	C16-Dihydroceramide	0.69	0.01
	(2R)-1-{[(2-Aminoethoxy)(hydroxy)phosphoryl]oxy}-3-hydroxy-2-propanyl (9Z)-9-tetradecenoate	0.72	0.01
19X_Manure_vs_Salt (13)	(2R)-3-({[(2S)-2,3-Dihydroxypropoxy](hydroxy)phosphoryl}oxy)-2-hydroxypropyl (9Z,12Z)-9,12-octadecadienoate	-0.4	ns
	N2-Dimethylguanosine	-0.61	0.035
	propionylcarnitine	-0.51	ns
	1,2,3,4-Tetrahydro-Î^{2}-carboline-3-carboxylic acid	-0.49	ns
	HHL	-0.27	ns
	Piracetam	-0.06	ns
	caroxazone	0.03	ns
	N6,N6,N6-Trimethyl-L-lysine	0.12	ns
	cis-3-Hexenyl phenylacetate	0.38	ns
	(1R)-6-Hydroxy-7-methoxy-1-methyl-1,2,3,4-tetrahydro-1-isoquinoliniumcarboxylate	0.38	ns
	(2E)-3-Phenyl-2-propen-1-yl 6-O-beta-D-arabinofuranosyl-beta-D-glucopyranoside	0.43	ns
	Astilbin	0.63	0.028
	Pindolol	0.84	0.0005
